# Multi-Channel Postoperative MRI and Deep Transfer Learning to Distinguish Glioblastoma Recurrence from Pseudo-Progression: A Proof-of-Concept Study

**DOI:** 10.3390/cancers18183015

**Published:** 2026-09-17

**Authors:** Ian D. Li, Cristina Correia, Choong-Yong Ung

**Affiliations:** Department of Molecular Pharmacology and Experimental Therapeutics, Mayo Clinic College of Medicine and Science, Rochester, MN 55905, USA; ianbubu2008@gmail.com (I.D.L.); correia.cristina@mayo.edu (C.C.)

**Keywords:** glioblastoma, deep transfer learning, 3D convolutional neural network, postoperative MRI, multiparametric MRI, pseudo-progression, tumor recurrence

## Abstract

Background/Objectives: Glioblastoma surveillance after surgery and chemoradiation remains challenging because MRI findings of tumor recurrence can overlap with pseudo-progression, treatment-related effects, and postoperative tissue changes. Methods: We developed a seven-channel postoperative MRI framework using a deep learning transfer method to support non-invasive glioblastoma treatment-effect assessment. The model used T1 contrast-enhanced, FLAIR, T1, RSI-Cell, ADC, T2, and cerebral blood flow volumes from 124 postoperative glioblastoma patients (164 MRI timepoints) as input. Images were processed using a 3D ResNet18 encoder pretrained on 588 postoperative glioma samples and fine-tuned using task-specific classification heads. The cohort comprised 124 patients contributing 164 postoperative MRI timepoints, all acquired at 3T on scanners from a single vendor. Performance was evaluated with nested five-fold cross-validation stratified and assigned at the patient level, so that all timepoints from a given patient fell in one-fold and the training epoch was selected on an inner split rather than on the fold being reported. Because some of the clinical labels were incomplete, the number of evaluable timepoints differed by task (recurrence versus pseudo-progression, 164; MGMT, 99; short-term survival, 139). The whole procedure was repeated under three independent random seeds and results are reported as the mean and standard deviation across seeds. Results: The strongest clinical endpoint was recurrence versus pseudo-progression, where nested cross-validation across three random seeds gave a pooled out-of-fold AUC of 0.935 (SD = 0.014), area under the precision-recall curve of 0.973, balanced accuracy of 0.880, sensitivity of 0.917, and specificity of 0.843. No other endpoint reached reliable discrimination. Radiation decision reached an AUC of 0.658 (SD = 0.039), while MGMT promoter methylation (AUC = 0.532, SD = 0.082) and short-term survival (AUC = 0.514, SD = 0.027) were indistinguishable from chance. Conclusions: In this single-center proof-of-concept study, postoperative MRI successfully distinguished tumor recurrence from pseudo-progression. However, the models did not reliably predict the other three outcomes related to molecular status, treatment planning, and prognosis. Overall, the model learned imaging features specifically associated with recurrence, rather than a more general representation of the tumor that can predict many different clinical outcomes. These findings support technical feasibility for a single endpoint rather than clinical readiness, for which external multi-center validation is required.

## Simple Summary

Glioblastoma follow-up after surgery and chemoradiation is clinically difficult because postoperative MRI changes can represent either recurrent tumor or treatment-related pseudo-progression. This study tested whether a seven-channel postoperative MRI representation analyzed using a transfer-learning model could support non-invasive treatment-effect assessment. The model was trained on four clinical endpoints: recurrence versus pseudo-progression, MGMT methylation, radiation decision, and short-term survival. Only one of them produced a reliable result. The model separated recurrence from pseudo-progression reliably. These results suggest that physiologically enriched postoperative MRI may help triage difficult glioblastoma surveillance cases. The same imaging did not predict molecular status, treatment planning decisions, or survival, so the useful signal appears specific to the recurrence question. External validation remains necessary before clinical use.

## 1. Introduction

Glioblastoma is the most aggressive adult-type diffuse glioma in current central nervous system tumor classification [1] and it remains a recalcitrant disease to treat. Currently, accurate diagnosis and disease management require the integration of imaging, histopathology, molecular testing, and longitudinal clinical assessment [2]. The standard treatment in glioblastoma consists of maximal safe surgical resection followed by radiotherapy with concomitant and adjuvant administration of temozolomide [3]. In routine clinical practice, magnetic resonance imaging (MRI) is the primary non-invasive tool to monitor tumor location, size, enhancing burden, surrounding tissue edema, monitor surgical cavity changes, and assess treatment response and recurrence, while O6-methylguanine-DNA methyltransferase (MGMT) promoter methylation is a clinically important predictive and prognostic biomarker for benefit from alkylating chemotherapy [4].

Conventional MRI is indispensable for diagnosing suspected glioblastoma, aiding neurosurgical and radiotherapy planning, and monitoring surveillance. An MRI system uses multiple independent receiver pathways, known as channels, to simultaneously capture radiofrequency signals from the body. The signals from all channels are then combined to reconstruct the final MR image. These channels include contrast-enhanced T1-weighted imaging, which depicts blood–brain barrier disruption and enhancing tumor; T2-weighted and fluid-attenuated inversion recovery (FLAIR) imaging, which captures infiltrative non-enhancing abnormalities and edema; diffusion-weighted metrics such as apparent diffusion coefficient (ADC), which reflect water mobility and cellularity; and perfusion imaging, which provides indirect information about tumor vascularity. However, conventional MRI alone is often insufficient to accurately define recurrence after surgery and chemoradiation because treatment-related enhancement, ischemia, blood products, inflammatory changes, and radiation effects can have overlapping features [5,6,7,8].

Currently, the most clinically difficult scenario is the distinction between true tumor progression or recurrence and pseudo-progression (PsP) [9]. To address this challenge, the Response Assessment in Neuro-Oncology (RANO) criteria were developed to standardize response assessment in high-grade gliomas and have been updated to reflect evolving neuro-oncology practice [10]. Nonetheless, response assessment still requires integration of several factors such as imaging timing, steroid use, clinical and neurological status, treatment history, and serial MRI scans. In the clinic, pseudo-progression is particularly problematic because it can mimic recurrence soon after chemoradiation and may lead to undesirable consequences including premature treatment discontinuation, unnecessary biopsy or tissue re-resection, and delays in implementing appropriate salvage therapies [11]. This ambiguity creates an urgent need for identifying reproducible imaging biomarkers that can separate treatment effect from active disease in individual patients.

Challenges in postoperative assessment are further compounded by the rapidly evolving landscape of glioblastoma therapeutics. Beyond maximal safe resection and chemoradiation, several approaches are currently being investigated to improve therapeutic delivery across the blood–brain barrier, including nanoparticle-based drug delivery like nanoliposomal irinotecan, focused ultrasound, and radio-immunotherapy combinations [12]. Each of the new modalities introduces its own pattern of post-treatment imaging change, which widens rather than narrows the gap between what conventional MRI shows and what clinicians would like to know. Imaging biomarkers that separate treatment effect from active disease are therefore likely to become more important, not less, as the therapeutic landscape broadens.

Advanced MRI has been used to address this gap. This includes diffusion and perfusion markers that provide physiologic information in complementing conventional anatomy. Meta-analytic and systematic evidence supports the value of perfusion-weighted MRI for distinguishing recurrent tumors from treatment-related necrosis or treatment effect, although performance varies across acquisition protocols, thresholds, and reference standards [13,14]. In addition, restriction spectrum imaging (RSI) extends conventional diffusion modeling by estimating restricted water compartments that may better reflect tumor cellularity in cancer imaging applications [15]. These methods are biologically attractive for postoperative glioblastoma because recurrence, necrosis, edema, vascular proliferation, and treatment effect are expected to differ in cellularity and perfusion even when their conventional MRI appearances overlap.

Machine learning (ML) and artificial intelligence (AI) have advanced brain tumor MRI analysis. Early works emphasized radiomics, segmentation, and survival modeling using hand-engineered features, while modern approaches increasingly use convolutional neural networks, transformers, self-supervised learning, and foundation-model strategies [16,17,18,19]. Importantly, the growth of public resources and benchmarks such as TCGA-glioma MRI derivatives, BraTS, UCSF-PDGM, and UPenn-GBM have enabled reproducible model development for tumor segmentation, radiogenomic classification, and survival prediction [20,21,22,23]. These tools have improved automated tumor delineation and shown that MRI indeed captures clinically important molecular and prognostic signals. Nevertheless, most widely used AI benchmarks remain preoperative or segmentation-centered, and many clinically important postoperative treatment-effect questions remain underrepresented.

Several groups have directly addressed pseudo-progression or treatment response using radiomics or deep learning. For instance, transparency-guided ensemble CNNs have been tested for PsP versus true progression, and multiparametric radiomics has shown promising preliminary performance for treatment-response assessment [24,25]. More recent self-supervised and multimodal studies have combined MRI with clinical or radiotherapy-planning variables [26,27]. Automated treatment-response classification has also been explored in challenge settings using hybrid deep learning and radiomics features [28]. At the same time, radiogenomic prediction of MGMT methylation and survival prediction remain challenging, with recent foundation-model and transfer-learning studies generally reporting modest and site-sensitive performance for molecular markers and survival endpoints [29,30].

Important clinical challenges and gaps remain. First, postoperative glioblastoma cohorts are small, heterogeneous, and label-noisy compared with natural-image datasets. Second, many models are trained for one endpoint at a time, which can waste shared imaging information across diagnosis, treatment planning, and prognosis. Third, standard MRI-only models may miss physiologic signals present in advanced diffusion and perfusion channels. Fourth, impressive internal performance can be undermined by patient-level leakage, hidden scanner effects, class imbalance, and generalizability of models [31,32,33,34]. Finally, clinical adoption requires model accuracy and interpretability, meaning clinicians need confidence that a model is using tumor and peritumoral substrate rather than irrelevant anatomy, acquisition artifacts, or label proxies.

The present study addresses these gaps by developing a seven-channel, 3D postoperative MRI framework with deep transfer learning. Each patient is represented by T1 contrast-enhanced (T1ce), FLAIR, T1, RSI-Cell, ADC, T2, and cerebral blood flow (CBF) volumes. A 3D ResNet18 encoder pretrained on a larger postoperative glioma cohort provides the initialization for every endpoint, and one model is then fine-tuned per endpoint across diagnostic, treatment-planning, molecular, and prognostic outputs [35,36,37,38]. Our central hypothesis is that postoperative multiparametric MRI contains learnable treatment-effect signatures, especially for recurrence versus pseudo-progression, and that domain-matched pretraining can make model development tractable in a cohort of this size.

Our study shows that advanced postoperative MRI may be moved from passive surveillance toward non-invasive treatment-effect decision support. However, this study is not positioned as a replacement for tissue diagnosis, molecular testing, expert neuroradiology, or multidisciplinary tumor-board judgment. Instead, this proof-of-concept study evaluates whether a unified, physiologically enriched MRI representation can help triage high-stakes postoperative glioblastoma questions, with recurrence versus pseudo-progression as the primary near-term clinical target and other endpoints serving as secondary evidence that the learned representation captures clinically relevant biology of glioblastoma.

## 2. Materials and Methods

### 2.1. Study Design and Data Sources

This retrospective MR imaging AI study used the glioblastoma UCSD-PTGBM dataset, identified in the Methods as The Cancer Imaging Archive (TCIA) postoperative glioblastoma collection [39,40]. The cohort included 124 postoperative glioblastoma patients contributing 164 MRI timepoints with multiparametric MRI and available clinical metadata. For transfer learning, the encoder was pretrained on MU-Glioma-Post, a larger postoperative glioma collection containing 588 samples. The pretraining cohort was used only to initialize the encoder before fine-tuning on the target UCSD-PTGBM cohort.

The UCSD-PTGBM cohort [40] comprises post-operative glioblastoma patients imaged at a single institution. In the 164 timepoints analyzed here, all examinations were acquired at 3T on GE Medical Systems scanners (DISCOVERY MR750, n = 105; Signa HDxt, n = 79) and no other field strengths or vendors were used. Clinical metadata included age, sex, MGMT promoter methylation status, IDH status, extent of resection, radiation decision, chemotherapy type, follow-up, and overall survival. These metadata were used to construct binary labels for the downstream prediction tasks. Patients with missing required MRI sequences were excluded before cross-validation splitting to ensure that all model inputs contained the same seven-channel structure. The analyzed cohort was derived by selecting patients with a primary diagnosis of glioblastoma who had undergone surgery and who were not IDH-mutant. This yielded 124 unique patients contributing 164 postoperative MRI timepoints, since a subset of patients was imaged at more than one postoperative timepoint. The timepoint is the unit of model input, whereas the patient is the unit of cross-validation assignment, and both counts are reported throughout to avoid ambiguity. Regarding molecular confirmation, no patient in the analyzed cohort carried a recorded IDH mutation. Patients with a documented IDH-wildtype result were retained, as were patients for whom IDH testing was not recorded, the latter on the basis of a histological glioblastoma diagnosis consistent with the WHO 2021 classification. The patient proportion in each category is given in Table 1. Patients recorded as IDH-mutant were excluded.

Two metadata inconsistencies were reviewed. First, the number_of_surgeries field was coded as 0 in 13 patients despite inclusion in a postoperative/post-procedural imaging cohort. These entries were retained and reported as “0 recorded” rather than interpreted as true absence of surgery, because eligibility was based on diagnosis, imaging availability, IDH status, and postoperative timepoints rather than on this field alone. Secondly, 12 biopsy-only cases were retained. Biopsy is a clinically valid surgical procedure in glioblastoma when maximal resection is unsafe or not feasible, and retaining these cases better reflects real-world postoperative/post-procedural surveillance. Extent-of-resection and number-of-surgeries metadata are therefore reported descriptively and were not used as primary outcome labels.

Cohort composition was verified directly against the clinical metadata. An initial audit showed that selecting every patient with usable imaging admitted cases inconsistent with the intended cohort, including IDH-mutant tumors and non-glioblastoma histology, which under the WHO 2021 classification are distinct entities. Inclusion was therefore applied explicitly: a primary diagnosis of glioblastoma, WHO grade 4, and absence of a recorded IDH mutation. Patients with IDH-mutant tumors, with non-glioblastoma histology, or with a grade below 4 were excluded before any model was fitted, and all analyses reported here use this filtered cohort.

### 2.2. MRI Preprocessing

The analysis used de-identified imaging and clinical metadata from public and/or project-approved retrospective datasets. All cross-validation and preprocessing steps were performed at the patient level. For each patient, the seven MRI sequences were resampled to a common voxel grid and aligned to create a channel-consistent 3D volume. Intensities were normalized per modality using z-score normalization calculated on non-zero voxels, reducing scanner- and sequence-dependent intensity scale differences while preserving relative intratumoral and peritumoral contrast. The final input consisted of a tumor-centered 96 × 96 × 96 voxel crop generated from the union of enhancing and FLAIR-hyperintense regions. This crop was designed to retain the enhancing tumor, surgical/treatment-effect region, non-enhancing abnormality, and immediate peritumoral environment while limiting computational burden for 3D training.

The seven channels were ordered as T1ce, FLAIR, T1, RSI-Cell, ADC, T2, and CBF. RSI-Cell was treated as a diffusion-derived cellularity-sensitive input channel, ADC as a conventional diffusion marker, and CBF as a perfusion marker. This representation was chosen to combine conventional postoperative anatomy with physiologic information relevant to vascularity and cellularity, two biological dimensions expected to differ between active tumor and treatment effect.

The physiologic channels, such as RSI-Cell, ADC and the perfusion maps, are distributed as derived volumes within the UCSD-PTGBM collection and were read directly as model inputs, with no further processing beyond the resampling and normalization described above. RSI-Cell is the restriction spectrum imaging cellularity map, which the collection derives by fitting a multi-compartment model to the multi-shell diffusion data and extracting the signal fraction of the most restricted compartment, the component most closely associated with dense tumor cellularity. ADC is the standard mono-exponential diffusion fit. The UCSD-PTGBM protocol includes both arterial spin labeling (ASL) and dynamic susceptibility contrast (DSC) perfusion acquisitions. In the present proof-of-concept analysis, the CBF input channel was populated by the available quantitative perfusion map distributed for a given scan: some timepoints contributed ASL_CBF, whereas others contributed CBF_svd derived from DSC perfusion. Thus, the seventh input channel should be interpreted as a pooled quantitative perfusion channel rather than a single homogeneous acquisition modality. This modality heterogeneity was not used for label construction and was not modeled as a covariate. But it is explicitly acknowledged as a limitation and should be standardized or harmonized in future external validations. Because all seven channels were resampled into the common image space before stacking, this channel structure preserved the intended anatomical alignment while combining complementary anatomical, diffusion, and perfusion information.

### 2.3. Clinical Prediction Tasks

Four binary tasks were defined and grouped into diagnostic, treatment-planning, and prognostic categories. The diagnostic tasks were Task 1, recurrence versus pseudo-progression, and Task 2, MGMT promoter methylation status. The treatment-planning task was Task 4a, radiation therapy decision classified as administered versus not administered, restricted to the 128 timepoints scored as recurrence, since the question of whether to irradiate only arises once recurrence is suspected. The prognostic task was Task 5, short-term survival, defined on overall survival rather than progression-free survival: a timepoint is positive if the recorded interval from that scan to death is shorter than 18 months and negative if it is 18 months or longer. Timepoints without a recorded date of death could not be labeled and were excluded rather than censored, which biases the negative class toward patients known to have died later and is revisited in Section 4. Treatment outcome, numbered Task 3, was removed because its label was the arithmetic inverse of the Task 1 label. Radiation-associated benefit, numbered Task 4b, was removed because it was conditioned on survival and structurally underpowered. The four retained endpoints keep their original numbers, so they appear as Tasks 1, 2, 4a and 5.

A sixth task, focused on treatment outcome, was also excluded because its label was the arithmetic inverse of the Task 1 label and the task therefore duplicated Task 1 rather than testing an independent endpoint.

The construction of the Task 5 label requires an explicit caveat. Short-term survival was derived from the recorded interval between the postoperative scan and death, so a timepoint entered the task only if a date of death was available. Patients who were alive at last follow-up carried no death date and were therefore excluded rather than treated as censored long survivors. Task 5 consequently discriminates earlier from later death among patients known to have died, and it does not estimate survival in the cohort as a whole. Because patients who survive longest are the most likely to be alive at last contact, the long-survival class is depleted of exactly the patients it should contain, and the reported performance for this endpoint should not be read as a survival model. A proper analysis would use last-follow-up dates to censor living patients and a time-to-event framework rather than a binary split; this is identified in Section 4 as required work.

These four endpoints are deliberately heterogeneous and are not clinically equivalent. Task 1 is a direct radiological diagnosis, Task 2 is a molecular property that imaging can only infer indirectly, Task 4a depends partly on clinician behavior and treatment access as well as on tumor biology, and Task 5 is a downstream prognostic outcome influenced by many non-imaging factors. They are grouped here because they share an imaging substrate, not because they are interchangeable, and the strength of evidence therefore differs by task. Task 1 is treated as the primary endpoint and the remaining tasks are secondary and exploratory.

Label availability differed by endpoint, and each task was evaluated only in patients with a recorded label. The resulting class distributions are given in Table 2. Two endpoints are notably reduced relative to the full cohort: MGMT status was recorded in 99 of 164 timepoints, and a date of death permitting the survival label in 139 of 164 timepoints. Results for these endpoints should therefore be read as exploratory and are more susceptible to small-sample variance than the Task 1 result.

### 2.4. Network Architecture and Transfer Learning

One model was trained for each endpoint. The backbone was a MONAI 3D ResNet18 encoder configured for volumetric input with seven channels [36,38], producing a 512-dimensional feature vector, followed by a two-layer multilayer perceptron with dropout probability 0.3 and a single binary output. Every endpoint used this identical architecture and the identical pretrained initialization. However, the models were fine-tuned separately and share no weights after initialization. The endpoints share the pretrained starting point, not a jointly optimized representation.

The encoder was initialized using weights pretrained on the MU-Glioma-Post dataset. During fine-tuning on UCSD-PTGBM, all encoder layers were unfrozen, allowing the representation to adapt to the target cohort while retaining postoperative glioma-specific initialization. The rationale for this strategy was that postoperative glioma MRI shares low- and mid-level features across cohorts, including surgical cavity morphology, enhancement patterns, edema topology, diffusion/perfusion heterogeneity, and spatial relationships between enhancing and non-enhancing disease.

One practical constraint of the pretraining step is that MU-Glioma-Post distributes only conventional sequences, so the pretraining encoder was configured from three input channels (contrast-enhanced T1, FLAIR, and pre-contrast T1) rather than the seven channels used on the target cohort. Transfer learning therefore initialized the shared convolutional blocks that are common to both configurations, while the first-layer weights corresponding to the four physiologic channels (RSI-Cell, ADC, T2, and CBF) were initialized randomly and learned entirely during fine-tuning. Pretraining consisted of 30 epochs of binary classification on the MU-Glioma-Post timepoints, and the encoder state retained was the one that maximized validation AUC during that run.

### 2.5. Training and Regularization

For reproducibility, the complete training configuration was as follows. Each model was a MONAI 3D ResNet18 accepting a seven-channel 96 × 96 × 96 volume and producing a 512-dimensional embedding, followed by a two-layer multilayer perceptron with dropout and a single binary output. Fine-tuning used AdamW [41] with a learning rate of 3 × 10^−4^ and weight decay of 5 × 10^−4^, applied uniformly to the encoder and the head. The schedule was a linear warmup over the first 10 epochs followed by cosine annealing with warm restarts (T_0 = 20, T_mult = 2). The objective was label-smoothed binary cross-entropy with smoothing 0.05 and a positive-class weight set inversely to label prevalence, so that patients without a label for a given endpoint were excluded from that endpoint entirely. Volume-level MixUp was applied with alpha = 0.1 for all tasks except MGMT, where alpha = 0.3 was used together with a higher dropout of 0.5 and freezing of the early encoder blocks, reflecting the smaller number of labeled cases for that endpoint. Training used a batch size of 4 with gradient accumulation and gradient-norm clipping, mixed precision on a single NVIDIA A100 GPU in Google Colab, and a maximum of 120 epochs with early stopping after 30 epochs without improvement. Every endpoint was trained under three random seeds (42, 123, 7) and results are reported as the mean and standard deviation across seeds. The identical configuration was applied to every cross-validation fold, and no per-fold hyperparameter search was performed.

Data augmentation included random flips along all three spatial axes, random affine transformations with rotation up to 10 degrees and scaling factors between 0.9 and 1.1, additive Gaussian noise (σ = 0.01), and volume-level MixUp (alpha = 0.1; alpha = 0.3 for the MGMT task) [42]. These augmentations were selected to mitigate overfitting risk in a limited postoperative cohort and encourage smoother decision boundaries while maintaining clinically plausible anatomical variation.

### 2.6. Safeguards Against Data Leakage and Class Imbalance

We implemented several strategies to prevent data leakage: (i) To prevent patient-level leakage, all cross-validation folds were constructed at the patient level: every scan belonging to a given patient appeared in exactly one fold. This prevents the model from memorizing patient-specific anatomy across the train-test boundary. Stratification was performed across the retained task labels when feasible so that label proportions were preserved in every fold. (ii) To prevent temporal leakage, task labels were derived exclusively from clinical metadata recorded at or after the time of the post-operative MRI scan. Recurrence status came from the negative-cases list described in Table 1. MGMT status, radiotherapy delivery and progression-free-survival came from the clinical metadata review, not from features encoded in the input volume. (iii) The model never sees future scans during training. The Task 1 labels were taken directly from the UCSD-PTGBM distribution and were not re-adjudicated by us. The collection publishes a list of negative cases identifying the timepoints its providers classified as treatment effect, pseudo-progression or radiation necrosis. Those timepoints form the negative class, and every remaining timepoint in the analyzed cohort forms the positive class. The positive class is therefore defined by exclusion rather than by positive adjudication, which is why it is the larger group at 128 of the 164 timepoints. We performed no independent histopathological or radiological review, applied no RANO scoring of our own, and excluded no timepoint on grounds of label ambiguity. The definitions that follow are those published by the dataset providers rather than criteria applied in this study: recurrence corresponds to histopathologically confirmed tumor progression or unequivocal radiographic progression per RANO criteria on at least two consecutive follow-up scans. Pseudo-progression corresponds to a transient enhancement increase within the first 6 months of concurrent temozolomide-radiation that subsequently stabilized or regressed without change in therapy. (iv) To reduce the effect of class imbalance, especially in Task 1, we used class-weighted loss and report AUC, balanced accuracy, sensitivity, and specificity rather than raw accuracy. Task 1 included 128 recurrence and 36 pseudo-progression timepoints; because pseudo-progression was the minority class, patient-level stratification was used when feasible. The small number of pseudo-progression cases remains a limitation.

### 2.7. Evaluation and Statistical Analysis

All tasks were evaluated with patient-level stratified five-fold cross-validation. Stratification was performed by task label when feasible, and folds were constructed at the patient level to prevent leakage across scans from the same patient. Model performance was summarized by area under the receiver operating characteristic curve (AUC), balanced accuracy, sensitivity, and specificity. Sensitivity and specificity were calculated at the optimal Youden index operating point within each fold [43]. Fold-level metrics were averaged across folds, and out-of-fold predictions were used to generate aggregate ROC curves. The project methods specify that 95% confidence intervals were estimated with 1000 patient-level bootstrap replicates.

Discrimination was summarized with AUC, balanced accuracy, sensitivity, and specificity. Because several endpoints are class-imbalanced, AUC and balanced accuracy were preferred over raw accuracy. Per-class precision, recall, and F1 score, precision-recall curves, calibration assessment, decision-curve analysis, and formal between-model comparison using the DeLong test were not performed in the present study; these are identified in Section 4 as required next steps.

The results reported in Section 3 come from a nested design in which each outer training set is split again, the epoch is chosen on the inner split, and the outer fold is scored once with the selected weights. For every endpoint, we report the optimism gap, defined as the mean AUC on the inner selection split minus the mean AUC on the outer test folds. A large positive gap means performance on the selection split did not carry over to data the model had never been scored on. Because several endpoints depend on variables in the metadata rather than on the scan, each was also fitted with a clinical comparator: a logistic regression on age, sex, extent of resection, number of surgeries, MGMT status, radiotherapy, bevacizumab exposure and first-line chemotherapy, trained and tested on exactly the patients and folds used by the imaging model. Chemotherapy variables were withheld from the comparator for the radiation endpoint, for the reason given in Section 3.4. Out-of-fold predictions were averaged across the three seeds so that each timepoint contributes once; the two models were compared with the DeLong test [44], and a logistic model holding both scores was used to test whether the imaging score retained an independent coefficient.

To support interpretability, Grad-CAM was applied to the final convolutional block of the shared encoder [45]. Attention maps were overlaid on resampled tumor-centered model-input slices and qualitatively reviewed for anatomic plausibility. Because the displayed underlays are cropped and resampled for visualization, they should be interpreted as localization aids rather than native-resolution diagnostic T1ce images. The specific goal was to confirm that task predictions were driven by tumor and peritumoral regions rather than distant brain tissue, skull, cropping artifacts, or scanner-related features.

## 3. Results

### 3.1. Overview of Model Development Pipeline

**Figure 1:**
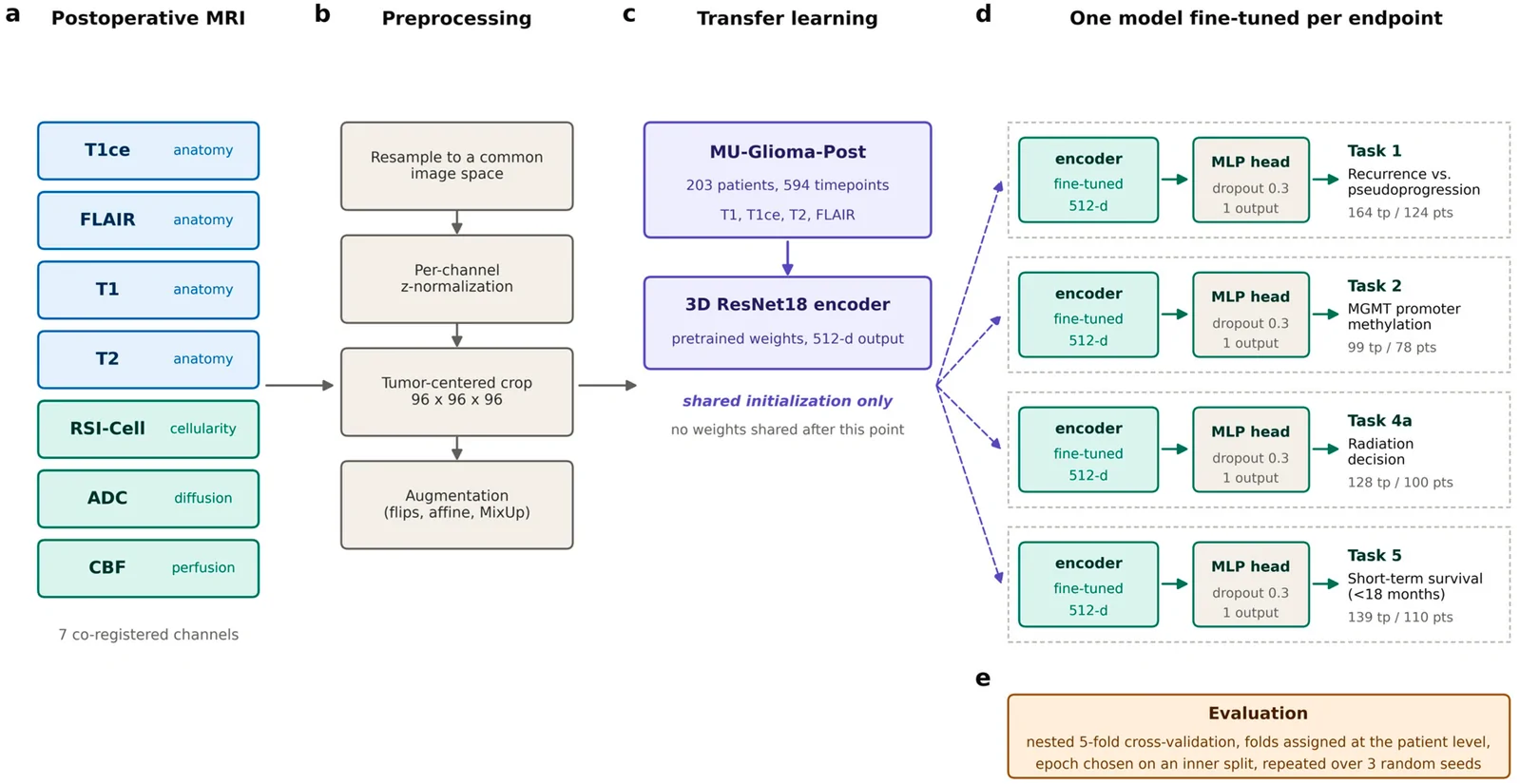
Study design and deep transfer-learning framework for postoperative glioblastoma treatment-effect assessment. a. Postoperative MRI. Seven co-registered postoperative MRI channels—T1ce, FLAIR, T1, RSI-Cell, ADC, T2, and CBF were generated for each patient and represented as tumor-centered 3D volumes. b. Preprocessing. This stage involves resampling to a common image space, per-channel z-normalization, tumor-center crop, and augmentation. c. Transfer learning. After preprocessing, modality normalization, tumor-centered cropping, and data augmentation, the seven-channel input was passed through a MONAI 3D ResNet18 encoder pretrained on the MU-Glioma-Post postoperative glioma cohort. d. One model fine-tuned per endpoint. One model was fine-tuned per endpoint from this common initialization, each producing a 512-dimensional embedding and a single binary output. The endpoint-specific models spanned diagnostic, treatment-planning, molecular, and prognostic endpoints: recurrence versus pseudo-progression, MGMT promoter methylation, radiation decision, and short-term survival < 18 months. e. Evaluation. Model performance was evaluated using patient-level stratified nested five-fold cross-validation across three random seeds. Grad-CAM visualization demonstrated tumor and peritumoral attention, supporting the biological plausibility of the learned postoperative MRI representation. ADC: apparent diffusion coefficient; CBF: cerebral blood flow; FLAIR: fluid-attenuated inversion recovery; MGMT: O6-methylguanine-DNA methyltransferase; RSI-Cell: restriction spectrum imaging cellularity map; T1ce: contrast-enhanced T1-weighted imaging.

We developed a transfer-learning framework to evaluate whether postoperative seven-channel MRI could support non-invasive glioblastoma treatment-effect assessment across available clinical endpoints. The overall study design included standardized preprocessing of postoperative MRI volumes, generation of seven co-registered imaging inputs: contrast-enhanced T1-weighted imaging, FLAIR, pre-contrast T1-weighted imaging, RSI-cellularity maps, ADC, T2-weighted imaging, and cerebral blood flow (CBF) maps, as well as supervised training of one three-dimensional (3D) ResNet-based model per endpoint from a common pretrained initialization. Table 3 summarizes the patient cohorts, MRI channels, and AI models used and Figure 1 provides an overview of the model pipeline and distinct classification tasks used.

**Figure 2:**
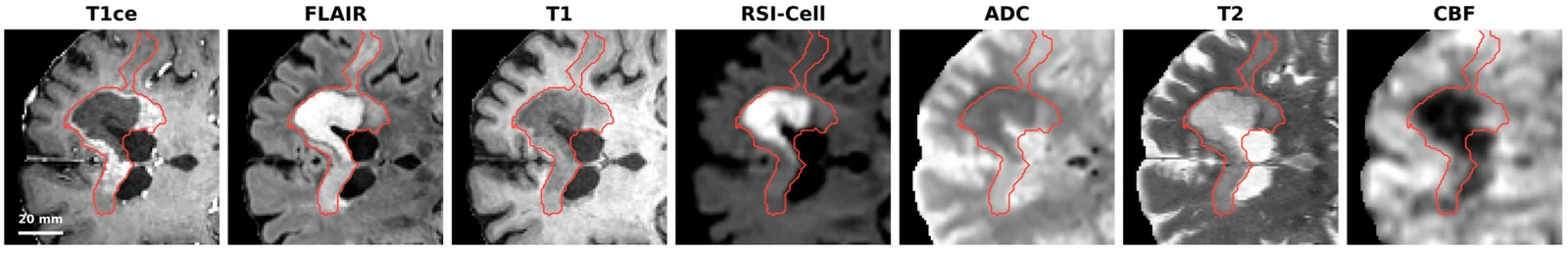
The seven co-registered channels that form a single model input. One representative postoperative timepoint is shown as a 96 × 96 × 96 tumor-centered crop, displayed at the axial slice with the largest tumor cross-section. Each panel is one channel of the same volume, windowed independently at the 1st and 99th percentiles, and the red outline is the tumor segmentation used to center the crop. Intensities are z-normalized within the brain mask, so panels are not comparable on an absolute scale. Scale bar 20 mm.

Representative seven-channel postoperative MRI inputs with respect to each classification task are shown in Figure 2. For each patient, the seven modalities (T1ce, FLAIR, T1, RSI-Cell, ADC, T2, and CBF) were resampled to a common voxel grid and spatially co-registered to a channel-consistent space, then cropped around the tumor region, as detailed in Methods (MRI preprocessing).

To improve feature learning in the setting of a limited postoperative glioblastoma cohort, the encoder was initialized using transfer learning from an independent postoperative glioma MRI dataset (MU-Glioma-Post cohort) and then fine-tuned on the UCSD-PTGBM target cohort for four clinically relevant tasks: recurrence versus pseudo-progression (Task 1), MGMT promoter methylation status (yes or no) (Task 2), radiation decision (administered versus not administered) (Task 4a), and short-term survival (<18 months) (Task 5). Model performance was evaluated using patient-level nested cross-validation, with area under the receiver operating characteristic curve (AUC), accuracy, balanced accuracy, sensitivity, specificity, precision, F1-score, and Matthews correlation coefficient used to characterize discrimination across tasks. This design allowed assessment of both the primary treatment-effect endpoint and the broader hypothesis that seven-channel postoperative MRI can encode biologically and clinically meaningful features relevant to tumor recurrence, molecular phenotype, treatment response, and survival risk.

### 3.2. Seven-Channel MRI and Domain-Matched Pretraining Supported Recurrence-Versus-Pseudoprogression Classification but Did Not Generalize to Other Endpoints

**Figure 3:**
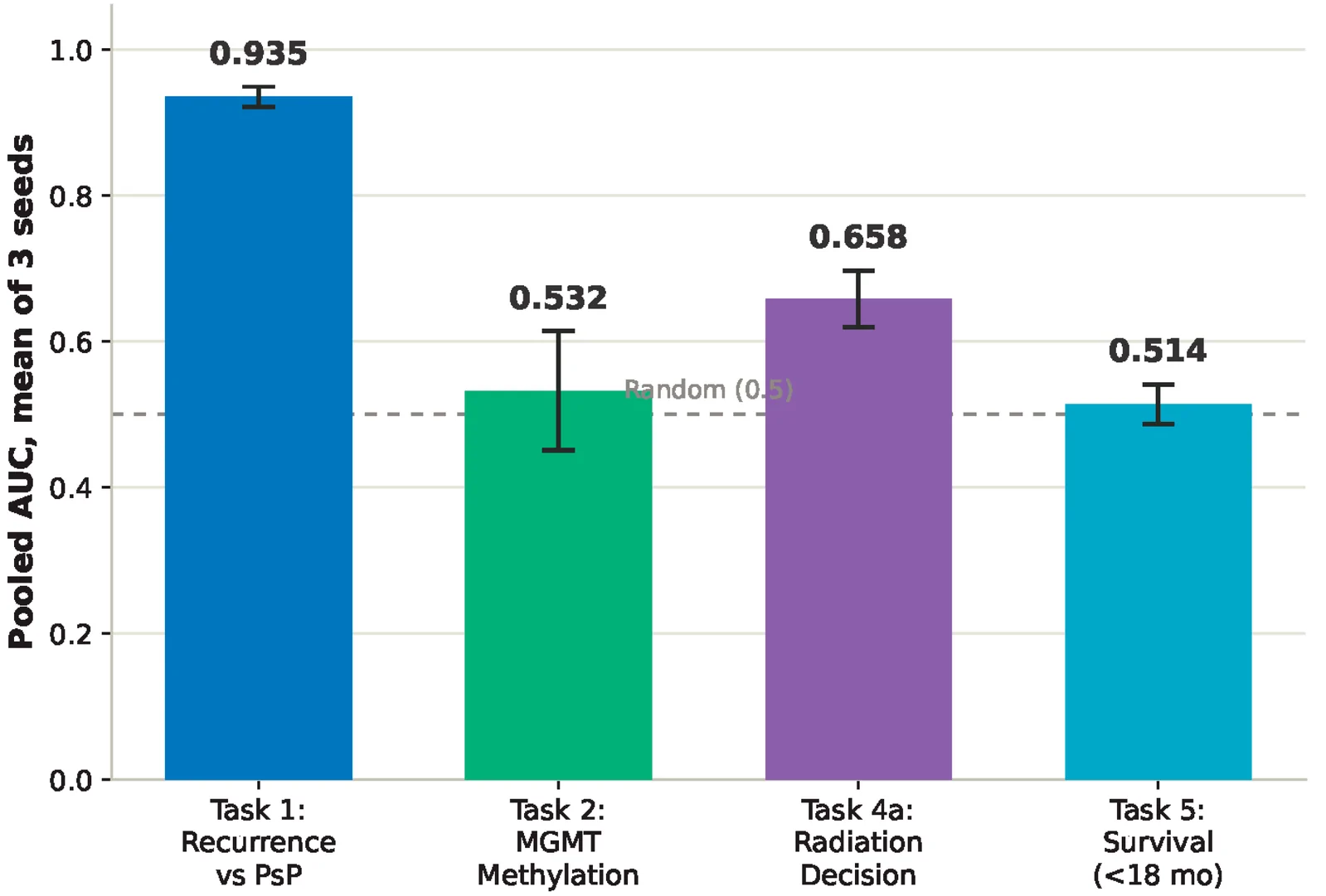
Overview of model performance across retained endpoints. The bar plot shows mean pooled AUC across three independent random seeds with SD error bars. Recurrence versus pseudo-progression was the only endpoint with reliable signal. The radiation decision was weak and confounded by treatment pathway, and MGMT methylation status and short-term survival were not above chance.

Figure 3 provides an overview of model performance for the four retained endpoints. We found the strongest classification task was Task 1, recurrence versus pseudo-progression. In nested five-fold cross-validation, with the training epoch selected on an inner split and each outer fold scored once, pooled out-of-fold performance was an AUC of 0.938, area under the precision-recall curve of 0.977, balanced accuracy of 0.882, sensitivity of 0.930, and specificity of 0.833. Per-fold outer AUCs were 0.952, 0.905, 0.965, 0.964, and 0.972, a narrow spread that indicates no single fold drives the aggregate. The patient-level bootstrap 95% confidence interval was 0.883 to 0.981. Reported per class, the model achieved precision 0.952, recall = 0.930, and F1 = 0.941 for recurrence (n = 128 timepoints), and precision = 0.769, recall = 0.833, and F1 = 0.800 for pseudo-progression (n = 36). Mean AUC on the inner selection split was 0.052 lower than on the outer test folds, so the nested procedure showed no residual selection optimism. Repeating the entire nested procedure under three independent random seeds gave a mean pooled AUC of 0.935 (SD 0.014). Our model therefore indicates strong separation between recurrent tumors and pseudo-progression across a wide range of performance thresholds. Overfitting was limited through several safeguards described in Methods: patient-level cross-validation with no scan shared across folds, transfer-learning initialization from the MU-Glioma-Post cohort, dropout layer in each task head, weight decay, label smoothing, and volume-level data augmentation.

To test whether domain-matched pretraining contributes to this result rather than merely being asserted, Task 1 was repeated under identical folds, seeds, and hyperparameters with the encoder randomly initialized. Pooled out-of-fold AUC fell from 0.938 to 0.924 and balanced accuracy from 0.882 to 0.848, but the informative difference lies in the minority class: specificity fell from 0.833 to 0.750 and recall for pseudo-progression fell by the same margin. Per-fold AUCs were also markedly less stable without pretraining, spanning 0.865 to 0.990 compared with 0.905 to 0.972 with it. Pretraining on an independent postoperative glioma cohort therefore improves the endpoint that matters clinically, correct identification of pseudo-progression, and roughly halves fold-to-fold variance, while leaving overall discrimination only modestly changed. This is consistent with the pretrained encoder supplying general postoperative tissue features that the target cohort cannot learn reliably from its 36 pseudo-progression cases alone. Results for both arms are given in Table 4.

A second ablation isolates the contribution of the physiologic channels. Task 1 was repeated under identical folds, seeds, hyperparameters and pretrained weights, with RSI-Cell, ADC and CBF held at zero throughout training and testing so that the model saw only conventional anatomy. Masking rather than a narrower first convolution was used so that both arms begin from exactly the same pretrained initialization. Pooled out-of-fold AUC fell from 0.935 (SD 0.012) to 0.864 (SD 0.013). The difference of 0.071 is more than five times the spread across seeds, and every seed shows the same direction.

Taken together, the results from the two ablation experiments summarized in Table 4 and Table 5 separate the two design choices that could account for Task 1 performance. Removing the domain-matched pretraining costs 0.014 AUC and removing the three physiologic channels costs 0.071. This suggests that the physiologic channels account for about five times as much of the result as the initialization does. The central claim of this study, that carrying diffusion- and perfusion-derived maps as native input channels rather than relying on anatomy alone improves the separation of recurrence from pseudo-progression, is therefore supported by direct experimentation. It also explains why the four conventional sequences that dominate public post-treatment glioma collections may be insufficient for this question.

The Task 1 performance is consistent with the known imaging physics, that is, true recurrence exhibits elevated cellularity (high RSI-Cell), restricted diffusion (low ADC), and neoangiogenesis-driven hyperperfusion (high CBF), whereas pseudo-progression shows treatment-related edema and blood–brain barrier disruption without true tumor cellularity [15,46,47]. These three advanced MRI sequences provide complementary biophysical contrast that is largely absent from conventional T1/T2/FLAIR-only pipelines, which may explain why prior imaging-only models report lower AUCs for this task. Because this endpoint is the most clinically actionable post-operative decision, this result should be interpreted as a strong internal validation signal that requires external validation and leakage audit before clinical translation. The retained endpoint-level performance metrics are summarized in Table 6.

### 3.3. Treatment Outcome Was Excluded and Short-Term Survival Was Not Reliably Predicted from Imaging

The treatment-outcome endpoint was excluded. Its label was constructed as the arithmetic inverse of the recurrence label, so the task was operationally identical to Task 1 rather than an independent endpoint, and reporting both would have counted one experiment twice.

Short-term survival (Task 5), defined as death within 18 months, reached a pooled out-of-fold AUC of 0.514 (SD = 0.027) across three seeds, with balanced accuracy 0.514, sensitivity 0.636, and specificity 0.391. Per-seed values were 0.498, 0.500, and 0.546. This endpoint is not distinguishable from chance. Fitted on the same patients and the same folds, a logistic regression on age, sex, extent of resection, number of surgeries, MGMT status and treatment exposure reaches 0.632, and adding the imaging score to that model changes nothing (*p* = 0.81). The prognostic information available in this cohort is carried by the recorded clinical variables rather than by the postoperative scan. Calibration for this endpoint is reported in Section 3.8.

### 3.4. MGMT Was Not Predicted from Imaging, and Radiation-Related Labels Were Weak or Confounded

MGMT promoter methylation (Task 2) reached a pooled out-of-fold AUC of 0.532 (SD 0.082; per-seed 0.619, 0.518, 0.458). This level of performance is consistent with the broader radiogenomics literature, where imaging-only prediction of MGMT status has proven difficult and often site-sensitive [30,48,49]. The value obtained here does not separate from chance, so we report it as a negative result. Imaging-only MGMT prediction is not supported by this cohort, and tissue-based molecular testing remains necessary.

Radiation decision (Task 4a) reached a pooled AUC of 0.658 (SD = 0.039), the only secondary endpoint above chance. It reached 0.406 with the largest optimism gap in the study at +0.360, but the more basic objection is that it was conditioned on survival, rested on 62 timepoints, and asked a causal question of a classifier. These labels are clinically important but more confounded than direct imaging diagnoses. The decision to administer radiation therapy reflects physician behavior, performance status, comorbidity, access, and treatment philosophy in addition to imaging. Radiation benefit is downstream of both imaging biology and non-imaging clinical factors, and an AUC below 0.5 with the largest optimism gap in the study is best read as an absence of imaging signal rather than as a weak one. Causal treatment-effect methods, external validation, and detailed clinical adjustment would all be needed before this endpoint can be revisited. Chemotherapy status alone separates irradiated from non-irradiated timepoints at an AUC of 0.989: 93 of the 94 irradiated timepoints have first-line chemotherapy recorded, against 2 of the 70 that were not irradiated, so the label is closer to an indicator of whether the patient received standard-of-care therapy than to a treatment-planning question. Radiotherapy also preceded the scan in 119 of the 121 timepoints carrying a radiation date, by a median of 147 days. What a model can recover from a post-radiation scan is therefore that radiation happened, not whether it should be given, and clinical variables predict the same label more accurately (0.800 against 0.690). We report this endpoint as detection of prior treatment and not as decision support.

### 3.5. Grad-CAM Localized Model Attention to Tumor and Peritumoral Regions

**Figure 4:**
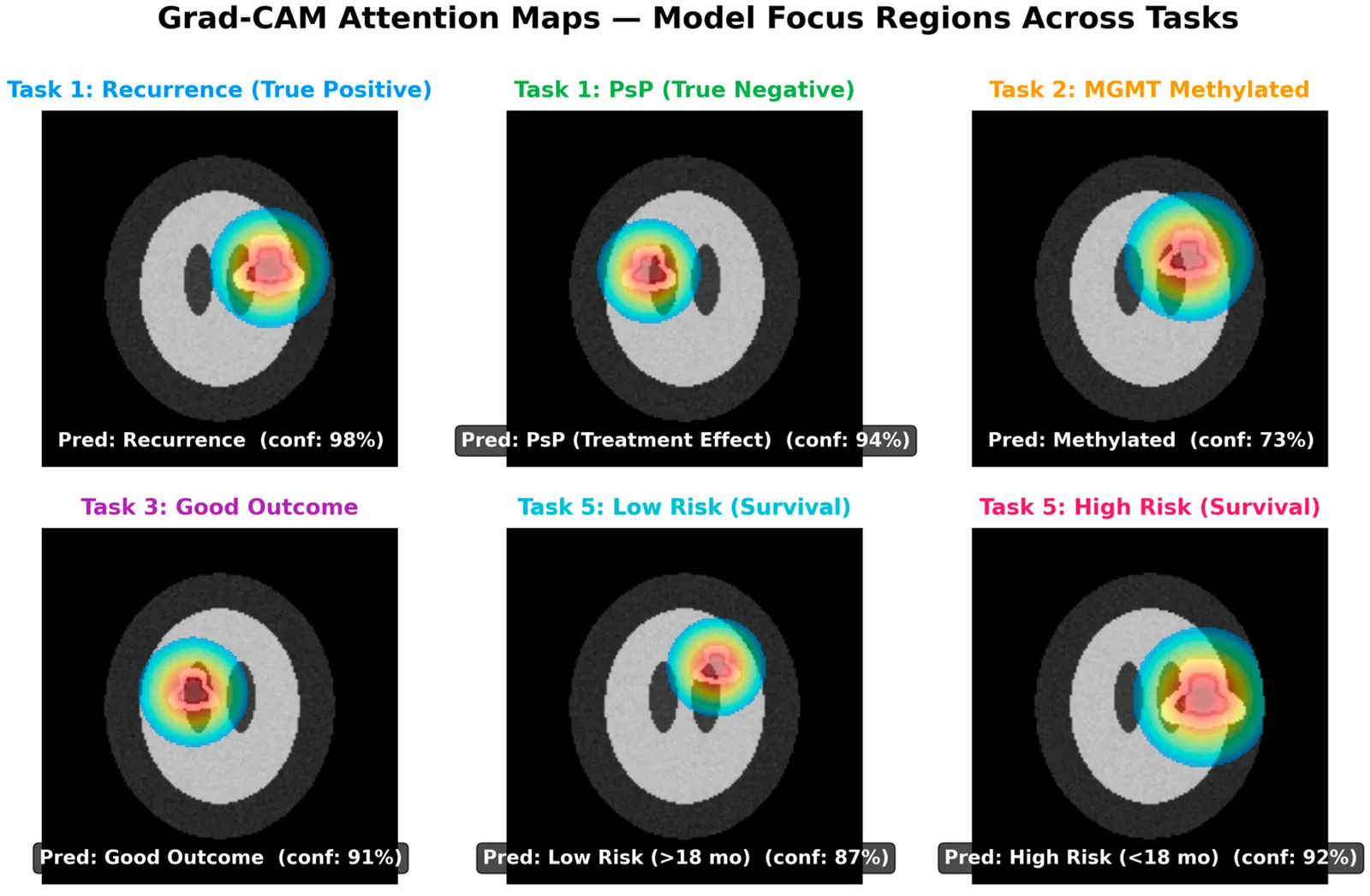
Representative Grad-CAM attention maps overlaid on resampled tumor-centered model-input slices. The underlays are cropped 96 × 96 × 96 model-input slices displayed for spatial localization and are not intended to represent native-resolution diagnostic T1ce images. Across representative examples, attention localizes to tumor and peritumoral regions rather than scanner artifacts or distant brain tissue.

Grad-CAM [45] maps demonstrated qualitatively plausible localization, and representative attention maps are shown in Figure 4. Across representative cases, model attention concentrated on the tumor and adjacent peritumoral region rather than distant brain structures or scanner artifacts. For recurrence versus pseudo-progression, attention was concentrated around the contrast-enhancing abnormality. For the endpoints that did not separate above chance, attention still fell on tumor and peritumoral regions, which shows that plausible-looking attention maps carry no guarantee of discriminative performance. Because Grad-CAM reflects gradient magnitude rather than a causal attribution, and because the overlays in Figure 4 are displayed on resampled tumor-centered model-input slices rather than native-resolution diagnostic T1ce images, these observations support biological plausibility but should not be interpreted as proof of causal feature use. The software used to generate these maps is listed in Supplementary Table S1.

### 3.6. Integrated Results Support a Task-Specific Imaging Signal Rather than a Unified Multi-Endpoint Framework

Taken together, the results do not support the hypothesis that one postoperative imaging representation is broadly useful across clinically diverse glioblastoma questions. One endpoint, recurrence versus pseudo-progression, is separated reliably. The remaining three are not: radiation decision reaches only weak discrimination, while MGMT methylation and short-term survival are indistinguishable from chance. The evidence presented here supports a single clinical application rather than a general-purpose postoperative representation.

A single pattern organizes these results. For each endpoint the nested design yields an optimism gap, the difference between mean AUC on the inner selection split and mean AUC on the outer test folds, and that gap orders the endpoints exactly as their test performance does. Recurrence versus pseudo-progression has a gap of −0.009, meaning outer-fold performance slightly exceeded the selection split. Every other endpoint has a large positive gap: radiation decision +0.137, short-term survival +0.154 and MGMT +0.189. A large positive gap is what fitting noise in a small selection set looks like, and the endpoints exhibiting it are precisely those that fail on held-out data. The nested design does not merely lower the headline figure; it separates one real effect from four spurious ones. These optimism gaps are summarized in Table 7.

### 3.7. Clinical, Volumetric, and Texture Comparisons Confirmed That the Imaging Signal Was Specific to Recurrence

An endpoint can look successful for two reasons: the imaging carries signal, or the label is already predictable from the recorded clinical variables. To separate them, every endpoint was refitted with a clinical comparator on the same patients and the same folds. Both models were compared on seed-averaged out-of-fold predictions, so each timepoint contributes once rather than three times. Because averaging predictions across seeds is a mild ensemble, these values run slightly above the per-seed means in Table 6.

Recurrence versus pseudo-progression is the only endpoint where imaging wins. The clinical model reaches 0.465, which is chance, against 0.961 for the imaging model (DeLong *p* < 0.001), and the imaging score keeps a strong coefficient when both scores sit in one model (*p* < 0.001). Age, extent of resection and molecular status carry nothing about whether a given enhancing lesion is tumor or treatment effect. That is what makes the Task 1 result an imaging finding rather than an artifact of case mix.

The other endpoints invert. Short-term survival is predicted better by the clinical variables (0.632) than by the imaging model (0.516), and the imaging term contributes nothing once clinical variables are present (*p* = 0.81). The question was pushed further for survival, because a comparison of two collapsed scores cannot detect an imaging feature that matters only in combination with a clinical one. Under the same patient-grouped cross-validation, clinical variables alone reach 0.630, adding the imaging score lowers this to 0.615, and adding every clinical-by-imaging interaction term lowers it further to 0.585. Performance falls monotonically as imaging information enters the model, which is what happens when the added variable is noise. MGMT methylation is weak in both models and indistinguishable between them (0.576 against 0.593, DeLong *p* = 0.84). Radiation decision is the one case where the imaging score keeps a coefficient (*p* = 0.019) while still losing to clinical variables (0.690 against 0.800, DeLong *p* = 0.048); Section 3.4 sets out why that increment should not be read as decision support.

A learned representation is not the only way to read a scan, and at this sample size it may not be the best one. We therefore repeated the two failing endpoints using measured features instead: eight volumetric quantities taken directly from the segmentation masks (enhancing tissue, non-enhancing core, FLAIR hyperintensity, resection cavity, their combinations, and two ratios) together with twelve physiologic summaries, the mean and standard deviation of ADC, CBF and RSI-Cell inside the enhancing region and inside the FLAIR hyperintensity. Twenty features were fitted by penalized logistic regression on the same patient-grouped folds.

For short-term survival, the measured features reached 0.550, above the 0.514 obtained by the network. Residual tumor burden therefore carries a little more prognostic information than the encoder recovered from the same images, which is the expected direction when 139 examples are used to fit a model with tens of millions of parameters. The gain does not survive contact with the clinical variables. Clinical features alone give 0.629 and adding the twenty imaging features gives 0.624. Under a Cox model with proper censoring, which uses 162 timepoints and 139 deaths rather than a binary cut, the ordering is the same: 0.562 for clinical variables, 0.538 for imaging, and 0.573 for both, a difference of 0.011 that is smaller than the spread across seeds.

For MGMT methylation, the measured features are at chance (0.502) and adding them to the clinical model lowers it from 0.610 to 0.561. Three independent approaches, a learned representation, a fusion of imaging and clinical scores including all interaction terms, and direct volumetric measurement, agree that postoperative MRI in this cohort carries no usable information about molecular status or survival. A fourth approach was added for completeness, since first-order summaries can miss fine spatial structure. Gray-level co-occurrence texture, higher-order intensity descriptors and tumor shape were computed at native resolution across five modalities and two regions, giving 134 candidate features with selection confined to the training folds. For MGMT, these reached 0.563, marginally above the network at 0.532, and adding them to the clinical model moved it from 0.610 to 0.623. For short-term survival, they reached 0.512 and lowered the clinical model from 0.629 to 0.569. Texture therefore carries slightly more MGMT-related information than the learned representation, consistent with the radiogenomics literature that uses such features, but not enough to change the conclusion for either endpoint.

### 3.8. Recurrence Predictions Required Recalibration but Showed Net Benefit in Decision-Curve Analysis

Discrimination and calibration are separate properties, and a model can rank cases well while assigning probabilities that do not match observed risk. Both were assessed on the same out-of-fold predictions, with calibration slope and intercept obtained by regressing outcome on the predicted log odds. Table 8 reports the results.

For recurrence versus pseudo-progression, the Brier score of 0.069 is low, but the calibration slope of 1.54 and intercept of 1.02 show a systematic bias: the model under-predicts. The reliability curve is monotone and under-confident in every populated bin. Cases assigned a mean probability of 0.33 recurred 75 percent of the time, and cases assigned 0.86 recurred 98 percent of the time. The cause is identifiable in the training objective rather than in the data. The positive-class weight was set to the inverse of label prevalence, which, for a cohort that is 78 percent recurrence, down-weights the majority class, and label smoothing of 0.05 pulls predictions toward 0.5 from both ends. Both choices raise balanced accuracy and neither alters the ranking of cases, so the AUC is unaffected, but the outputs should not be read as calibrated probabilities of recurrence. Recalibration on a held-out split is the standard remedy and is required before any probability from this model is shown to a clinician.

Despite that bias, the model is clinically useful under decision-curve analysis. Standardized net benefit exceeds both default strategies, treating every patient as recurrence and treating none, across the entire threshold range examined. At a decision threshold of 0.5, the model returns 0.701 against 0.561 for treating all, and at 0.8, where a clinician would demand high confidence before acting, it returns 0.518 against minus 0.098. Decision-curve analysis is insensitive to calibration drift of this kind because it depends on the ordering of cases relative to a threshold, which is the same quantity the AUC measures. This is the first evidence in the study that the model would change management favorably rather than merely separate classes.

The remaining endpoints corroborate the negative results from a different direction. Calibration slopes of 0.12 for MGMT methylation and 0.12 for short-term survival are close to zero, meaning the predicted log odds carry almost no information about the outcome, which is what a chance-level AUC implies and what the clinical comparator in Section 3.7 already indicated. The imaging-versus-clinical comparisons are summarized in Table 9.

## 4. Discussion

Prior machine learning works have demonstrated the feasibility of MRI-based treatment-effect classification. However, many of these studies generally remain limited by single-task designs. For instance, Liu et al. used transparency-guided ensemble CNNs in 84 GBM patients and reported 90.2% accuracy for pseudo-progression versus true progression [24]. Parekh et al. reported preliminary multiparametric radiomics performance with an AUC of 0.93 for pseudo-progression versus true progression in a small brain-tumor cohort [25]. A more recent self-supervised multimodal work by Gomaa et al. combined MRI, clinical variables, and radiotherapy-planning information and reported an AUC of 0.753 on an external test cohort [26]. Another recent stage-specific benchmarking study across 11 deep-learning model families reported accuracies around 0.70 to 0.74, emphasizing the intrinsic difficulty of the task and the need for standardized training and validation [27].

This study developed seven-channel postoperative MRI models with deep transfer learning for non-invasive glioblastoma treatment-effect assessment. The principal finding was strong internal discrimination of recurrence versus pseudo-progression (Task 1) under nested cross-validation, with a pooled out-of-fold AUC of 0.938 and balanced accuracy of 0.882. The same approach did not transfer to the other endpoints. Radiation decision (Task 4a) reached weak discrimination at 0.658, and MGMT methylation and short-term survival were indistinguishable from chance. The findings therefore support a narrower claim than the one originally advanced: postoperative multiparametric MRI contains a learnable representation relevant to distinguishing recurrence from treatment effect, but not to molecular phenotype, treatment-planning behavior, or survival in this cohort. The clinical value of a reliable tool in this setting is high because misclassifying pseudo-progression as recurrence can trigger unnecessary intervention or premature treatment change, whereas misclassifying recurrence as treatment effect can delay salvage therapy. Our model therefore reflects the biological value of seven-channel post-operative MRI, particularly RSI-Cell that estimates restricted water compartments, ADC that reflects water mobility and cellularity, and CBF on cerebral blood flow volumes. Our model may also reflect favorable internal cohort structure, strong preprocessing, or a label distribution that is more separable than external cohorts. Our study suggests that combining conventional MRI with diffusion- and perfusion-derived channels may capture treatment-effect biology more effectively than conventional anatomy alone.

We show that domain-matched pretraining can make small postoperative imaging cohorts more tractable. Pretraining on a related postoperative glioma cohort helps the encoder learn general tumor, edema, and surgical-cavity features before endpoint-specific fine-tuning, and the ablation in Table 4 quantifies that contribution as real but modest. The combination of diffusion-sensitive and perfusion-sensitive channels is biologically plausible for differentiating active tumors from treatment effect because recurrence is expected to show different cellularity and vascularity patterns from pseudo-progression or necrosis. That mechanism did not extend to the other endpoints. Short-term survival in particular reached only 0.514, which is what should be expected once the cohort is restricted to IDH-wildtype grade 4 disease, where prognosis is uniformly poor and the subgroup separations that imaging might detect have been removed by the inclusion criteria. The clinical comparator in Section 3.7 sharpens this. Where imaging failed, a handful of routinely recorded variables did better, and the imaging score added nothing on top of them. This was tested three ways rather than assumed. The representation is not weak in general; it is specific. It encodes the local tissue question it was asked, and it does not encode the patient-level factors that drive molecular status, treatment assignment and survival.

On the other hand, MGMT methylation (Task 2) is a molecular property measured from tissue, and imaging-only radiogenomic prediction is anticipated to be difficult. The observed value is not distinguishable from chance, and the seed-to-seed spread exceeds the distance from 0.5, so we report this endpoint as a negative result rather than as weak evidence of radiogenomic signal. This is consistent with the largest external evaluation of the question and with subsequent deep-learning evaluations. In the BraTS 2021 radiogenomics challenge, 420 models were trained across combinations of dataset, architecture, sequence and seed, and roughly 80 percent showed no significant difference from chance on external test accuracy, while the challenge-winning solution reached an external AUROC of 0.562 [48]. Saeed et al. similarly reported that extensive deep-learning evaluation found no reliable MRI signal for MGMT promoter methylation prediction [50]. Two features of the present setting make the task harder still. Those studies used preoperative imaging, whereas the tumor whose methylation status is being predicted here has already been resected, and our 99 evaluable timepoints are a fraction of the sample sizes used there. The radiation decision endpoint (Task 4a) should likewise be read as exploratory. It is not a pure imaging label: it reflects clinical decision-making, patient fitness, disease extent, treatment access and post-treatment care, and Section 3.4 shows that it is largely determined by whether the patient received standard-of-care therapy at all.

A recent segmentation-focused work has shown that pretrained backbone U-Net architectures, particularly ResNet-U-Net, improve glioma subregion segmentation and that T1Gd and FLAIR provide complementary information for active tumor/necrosis and edema delineation [51]. However, segmentation alone does not resolve the postoperative clinical problem of distinguishing recurrence from pseudo-progression. Our study extends this direction by using seven-channel postoperative MRI, including diffusion- and perfusion-derived physiologic channels, within a 3D transfer-learning framework to predict treatment effects and outcome endpoints at the patient level.

More work is still needed before the current model can be used in clinics. Independent external validation on a multi-institutional postoperative GBM cohort with harmonized labels for recurrence and pseudo-progression will be conducted. To facilitate clinical translation, a reader study comparing neuroradiologists alone versus neuroradiologists assisted by the model will be conducted, especially for Task 1. Also, for radiation-related tasks, we will incorporate clinical covariates and causal inference methods to separate physician selection from treatment effect in future work. Finally, we will perform prospective testing to evaluate how our model predictions alter management decisions, reduce unnecessary procedures, shorten diagnostic uncertainty, or improve patient outcomes.

Three aspects of this work differ from previously published approaches. First, most published postoperative models operate on conventional anatomical sequences alone; here, diffusion- and perfusion-derived physiologic maps (RSI-Cell, ADC, and CBF) are carried as native input channels alongside T1ce, T1, T2, and FLAIR, so that cellularity and vascularity information reaches the encoder directly rather than through hand-engineered features, and the ablation in Table 5 shows this is worth 0.071 AUC. Second, the same domain-matched initialization is reused across every endpoint, so a modest postoperative cohort supports several clinical questions without a separate pretraining run for each. Third, pretraining is performed on an independent postoperative glioma cohort rather than on natural images or preoperative scans, so the source and target domains share surgical cavity morphology and treatment-related change.

Additional limitations arise from the perfusion-channel and surgical metadata. The CBF channel pooled quantitative perfusion maps from two source types, ASL_CBF and DSC-derived CBF_svd, so future work should train and validate separate or harmonized perfusion inputs. The surgical metadata also contained 13 patients coded as having zero surgeries and 12 biopsy-only cases. These cases were retained and reported rather than excluded, but both variables should be audited against operative records in any prospective validation.

The contrast between the strong Task 1 result and the null MGMT and radiation-related results deserves direct comment rather than being presented as a simple gradient of difficulty. Part of the gap is plausibly biological: recurrence and pseudo-progression differ in cellularity and perfusion, which are precisely the properties that RSI-Cell, ADC, and CBF encode, whereas MGMT promoter methylation has no comparably direct imaging correlate and radiation decisions reflect clinician judgment as much as tumor biology. Part of it is a question of sample size, since the MGMT and survival endpoints were fitted on 99 and 139 timepoints, respectively, rather than the full 164. Two potential methodological explanations were examined directly. The first, leakage across cross-validation folds from patients contributing several postoperative timepoints, can be excluded: fold assignment was performed on patient identifiers with the timepoint suffix removed, so all studies belonging to one patient were confined to a single fold. A residual concern also attaches to the label definition, since pseudo-progression was defined partly by subsequent radiological stabilization, which introduces a degree of circularity between the label and the imaging evolution the model is asked to predict. Nested cross-validation has now been performed, and the measured optimism gap was negative, meaning performance on the outer folds slightly exceeded that on the inner selection split. Selection bias therefore does not account for the Task 1 result. What remains untested is generalization beyond this single institution, so an AUC of 0.938 should be read as an internal-validity estimate pending an external cohort.

Several limitations follow from the design and from the composition of the dataset. Radiotherapy was represented only as a binary record of whether treatment was administered; prescribed dose, fractionation, and target volume were not available, so dose-dependent treatment effect could not be modeled. Chemotherapy was likewise recorded only as first-line agent, and 75 patients had no chemotherapy record, so temozolomide exposure could not be treated as a covariate. Established prognostic variables including tumor location, residual tumor volume, and performance status were not present in the clinical metadata and therefore could not be included or adjusted for, which is a material limitation for the survival and treatment-outcome endpoints in particular. Label completeness also varied considerably: the survival label was assignable for 139 of 164 timepoints and MGMT status for 99 of 164, so the corresponding models were fitted on substantially smaller and possibly non-random subsets, and the survival result rests on only 21 patients surviving at least 18 months. Finally, all imaging came from a single institution and a single vendor at one field strength. This is favorable for protocol consistency but means that vendor and protocol robustness is entirely untested, and it is one reason the internal performance reported here should not be assumed to transfer.

Two further limitations concern how endpoints were defined rather than how the model was fitted. First, as described in Section 2.3, the survival endpoint is conditioned on death having been recorded, so it excludes living patients instead of censoring them. The resulting estimate applies to patients known to have died and cannot be extrapolated to the cohort, and a time-to-event analysis with proper censoring is required before any prognostic claim is made. Second, the recurrence versus pseudo-progression endpoint is more imbalanced than a first reading of the cohort suggests, with pseudo-progression the minority class by a wide margin. Balanced accuracy and AUC were chosen partly for this reason, but with few pseudo-progression cases distributed across five folds, each held-out partition contains only a small number of them, and per-fold estimates for this endpoint are correspondingly unstable.

## 5. Conclusions

This proof-of-concept study shows that seven-channel postoperative MRI combined with transfer learning separates recurrence from pseudo-progression with a pooled out-of-fold AUC of 0.935 across three random seeds. The study contributes a proof-of-concept framework for small datasets and non-invasive glioblastoma treatment-effect assessment. Three contributions distinguish this work from single-task studies. First, it combines conventional anatomical sequences with diffusion- and perfusion-derived physiologic channels (RSI-Cell, ADC, and CBF) in a single volumetric representation rather than relying on anatomy alone, and an ablation shows those channels account for 0.071 of the 0.935 AUC. Second, it reuses one domain-matched pretrained initialization across four clinically distinct endpoints, so a limited postoperative cohort supports several clinical questions. Third, it applies transfer learning from an independent postoperative glioma cohort, which is a closer domain match than natural-image or preoperative pretraining. Decision-curve analysis in Section 3.8 shows net benefit above both default strategies across the threshold range, so these results are evidence of technical feasibility and of potential clinical utility for one clinical question, not as evidence of clinical utility, and not as support for a general postoperative imaging representation. Three of four endpoints failed under nested evaluation, and the ordering of those failures by optimism gap indicates that their apparent performance under the earlier procedure reflected selection on the evaluation fold rather than imaging signal. A clinical-variable comparator fitted on the same folds puts the surviving endpoint in context: clinical variables are at chance for recurrence versus pseudo-progression while the imaging model is not, and they outperform imaging on survival and radiation while imaging adds nothing to them. The cohort was single-center and single-vendor, several endpoints had substantial missing labels, and no external cohort was tested. External multi-center validation, calibration and decision-curve analysis, and formal ablation of the remaining architectural components are each required before this framework can be considered for clinical decision support.

## Figures and Tables

**Table 1:** Clinical and imaging characteristics of the postoperative glioblastoma cohort. Figures are per patient (n = 124); these 124 patients contributed 164 postoperative MRI timepoints, which are the unit of model input. Missing values are reported explicitly because label availability differed by endpoint and determined the number of evaluable timepoints per task.

Characteristic	Value	Missing, n (%)
Age at scan, years	Median 57 (IQR 49–66; range 26–88)	0
Sex	Male 89 (71.8%); Female 35 (28.2%)	0
Extent of resection	Gross total 68 (54.8%) Subtotal 44 (35.5%) Biopsy only 12 (9.7%)	0
Number of surgeries	0 recorded: 13 (10.5%; retained as metadata inconsistency) 1: 80 (64.5%) 2: 26 (21.0%) 3: 5 (4.0%)	0
WHO grade	Grade 4: 124 (100%)	0
IDH status	Wild-type 82 (66.1%) Not recorded 42 (33.9%)	42 (33.9%)
MGMT promoter	Methylated 38 (30.6%) Unmethylated 40 (32.3%)	46 (37.1%)
Radiotherapy	Administered 77 (62.1%) Not administered 47 (37.9%)	0
First-line chemotherapy	Temozolomide 82 (66.1%) Lomustine 2 (1.6%)	40 (32.3%)
Bevacizumab	Yes 17 (13.7%) No 107 (86.3%)	0
Overall survival, days	Median 457 (IQR 242–610)	68 (54.8%)
Progression-free survival, days	Median 188 (IQR 46–348)	69 (55.6%)
Field strength/vendor	3T; GE (DISCOVERY MR750 70, Signa HDxt 54)	0

**Table 2:** Number of evaluable timepoints (with the corresponding number of unique patients in parentheses) and class distribution for each of the retained classification tasks. Timepoints without a recorded label for a given endpoint were excluded from that task only. Fold assignment was always performed on patients, so all timepoints from one patient remained within a single fold.

Task	Evaluable Timepoints, n (Patients)	Class Distribution	Excluded, n
Task 1: Recurrence vs. pseudo-progression	164 (124)	See Section 2.6	0
Task 2: MGMT methylation status	99 (78)	Methylated 47/Unmethylated 52 timepoints	65
Task 4a: Radiation decision	128 (100)	Administered 78/Not administered 50 timepoints	36
Task 5: Short-term survival (<18 months)	139 (110)	Short 87/Long 52 timepoints	25

**Table 3:** Summary of glioblastoma patient cohorts, MRI parameters, and AI model characteristics.

Characteristic	Value	Description
Target cohort	124 patients (164 timepoints)	Postoperative glioblastoma from UCSD-PTGBM/TCIA.
Pretraining cohort	588 samples	MU-Glioma-Post cohort used to initialize each endpoint model.
MRI channels	7	T1ce, FLAIR, T1, RSI-Cell, ADC, T2, and CBF.
Input volume	96 × 96 × 96 voxels	Tumor-centered crops after resampling and modality normalization.
Model backbone	3D ResNet18	MONAI implementation configured for seven input channels.
Cross-validation	5-fold cross-validation	Patient-level stratified cross-validation to reduce leakage risk.

**Table 4:** Effect of encoder initialization on Task 1 under identical folds, seeds, and hyperparameters. Both arms use nested cross-validation with epoch selection confined to an inner split.

Metric	Random Initialization	Pretrained Encoder
Pooled AUC (95% CI)	0.924 (0.855–0.976)	0.938 (0.883–0.981)
AUPRC	0.957	0.977
Balanced accuracy	0.848	0.882
Sensitivity (recurrence)	0.945	0.930
Specificity (pseudo-progression)	0.750	0.833
F1, recurrence	0.938	0.941
F1, pseudo-progression	0.771	0.800
Per-fold AUC range	0.865–0.990	0.905–0.972
Optimism gap (inner minus outer)	−0.027	−0.052

**Table 5:** Effect of removing the diffusion- and perfusion-derived channels from Task 1. Under this task, identical folds, seeds, hyperparameters and pretrained weights were used.

Metric	Conventional Anatomy	All Seven Channels	Difference
Pooled AUC, mean (SD)	0.864 (0.013)	0.935 (0.012)	+0.071
Per-seed AUC	0.846, 0.878, 0.867	0.924, 0.929, 0.951	-
Channels supplied	T1ce, FLAIR, T1, T2	Plus RSI-Cell, ADC, CBF	-

**Table 6:** Model performance using patient-level nested five-fold cross-validation across retained clinical tasks.

Classification Task	AUC	Balanced Accuracy	Sensitivity	Specificity
Diagnostic: Task 1—Recurrence vs. pseudo-progression	0.935 (0.014)	0.880	0.917	0.843
Diagnostic: Task 2—MGMT methylation status	0.532 (0.082)	0.526	0.546	0.506
Treatment planning: Task 4a—Radiation decision	0.658 (0.039)	0.597	0.607	0.587
Prognostic: Task 5—Short-term survival (<18 months)	0.514 (0.027)	0.514	0.636	0.391

**Table 7:** Endpoints ordered by optimism gap, the difference between mean AUC on the inner selection split and on the outer test folds. All values are pooled out-of-fold results across three random seeds. The ordering by gap matches the ordering by test performance.

Endpoint	Timepoints (Patients)	AUC, Mean (SD)	Optimism Gap	Interpretation
1. Recurrence vs. pseudo-progression	164 (124)	0.935 (0.014)	−0.009	Reliable
4a. Radiation decision	128 (100)	0.658 (0.039)	+0.137	Weak
5. Short-term survival	139 (110)	0.514 (0.027)	+0.154	Not above chance
2. MGMT promoter methylation	99 (78)	0.532 (0.082)	+0.189	Not above chance

**Table 8:** Calibration of the out-of-fold predictions. Perfect calibration corresponds to a slope of 1.00 and an intercept of 0.00. ECE is the expected calibration error over ten equal-width bins. Lower Brier and ECE are better.

Endpoint	AUC	Brier Score	Slope	Intercept	ECE
1. Recurrence vs. Pseudo-progression	0.961	0.069	1.54	1.02	0.117
4a. Radiation decision	0.690	0.223	0.55	0.33	0.102
2. MGMT promoter methylation	0.576	0.268	0.12	−0.12	0.175
5. Short-term survival	0.516	0.258	0.12	0.46	0.140

**Table 9:** Imaging model against a clinical-variable model on identical patients and folds. Predictions are averaged across three random seeds so that each timepoint contributes once. DeLong *p*-value compares the two AUCs. The final column is the Wald *p* for the imaging score in a logistic model that already contains the clinical score; a value above 0.05 means postoperative MRI adds nothing to what the clinical metadata already records.

Endpoint	n	Imaging	Clinical	Both	DeLong *p*	Imaging Term *p*
1. Recurrence vs. pseudo-progression	164	0.961	0.465	0.962	<0.001	<0.001
2. MGMT promoter methylation	99	0.576	0.593	0.619	0.84	0.67
4a. Radiation decision	128	0.690	0.800	0.838	0.048	0.019
5. Short-term survival	139	0.516	0.632	0.633	0.061	0.81

## Data Availability

The UCSD-PTGBM imaging dataset is available through The Cancer Imaging Archive [39,40]. The MU-Glioma-Post pretraining collection is available from its original public repository. Trained model weights, training and evaluation code, and the cross-validation fold definitions used in this study are available from the corresponding author on reasonable request.

## Supplementary Materials

The following supporting information can be downloaded at https://www.mdpi.com/article/10.3390/cancers18183015/s1: Table S1. Software, models, datasets and training configuration used in this study. References [52,53] are cited in Supplementary Materials.

## Abbreviations

The following abbreviations are used in this manuscript: ADC, apparent diffusion coefficient; AI, artificial intelligence; AUC, area under the receiver operating characteristic curve; CBF, cerebral blood flow; FLAIR, fluid-attenuated inversion recovery; GBM, glioblastoma; Grad-CAM, gradient-weighted class activation mapping; MGMT, O6-methylguanine-DNA methyltransferase; MRI, magnetic resonance imaging; PsP, pseudo-progression; RANO, Response Assessment in Neuro-Oncology; RSI, restriction spectrum imaging; T1ce, contrast-enhanced T1-weighted imaging.
