# Supplementary material for: Multi-Channel Postoperative MRI and Deep Transfer Learning to Distinguish Glioblastoma Recurrence from Pseudo-Progression: A Proof-of-Concept Study
Source: Cancers (Basel). 2026 Sep 17;18(18):3015. doi: 10.3390/cancers18183015 (PMC13606005; doi:10.3390/cancers18183015)
Supplement: Supplementary file 1 [file cancers-18-03015-s001.zip › cancers-4461254-supplementary.pdf]

Supplementary Materials

Supplementary Table S1. **Software, models, datasets and training configuration used in this study**. Configuration values are those actually used to generate the results reported here, verified against the training code.

Component	Type	Version or configuration	Reference or source
UCSD-PTGBM	Target dataset	124 patients, 164 postoperative timepoints, 7 channels	TCIA; Sci. Data 2026 [40]
MU-Glioma-Post	Pretraining dataset	203 patients, 594 timepoints, 4 conventional sequences	TCIA, DOI 10.7937/7K9K-3C83; Mahmoud et al., Sci. Data 2025, 12, 1847
Python	Language	3.13	python.org
PyTorch	Framework	2.6, automatic mixed precision	pytorch.org
MONAI	Framework	3D ResNet18 backbone	Cardoso et al. [36]
3D ResNet18	Encoder	7-channel 96 x 96 x 96 input, 512-dimensional embedding	He et al., CVPR 2016; Chen et al., Med3D, arXiv:1904.00625
Classification head	Model	Two-layer MLP, dropout 0.3, single binary output, one per endpoint	This study
AdamW	Optimizer	Learning rate 3e-4, weight decay 5e-4	Loshchilov and Hutter [41]
Learning-rate schedule	Training	Linear warmup over 10 epochs, then cosine annealing with warm restarts (T_0 = 20, T_mult = 2)	This study
Objective	Training	Label-smoothed binary cross-entropy, smoothing 0.05, positive-class weight set to inverse label prevalence	This study
MixUp	Augmentation	alpha = 0.1, and alpha = 0.3 for MGMT with dropout 0.5 and early blocks frozen	Zhang et al. [42]
Training control	Training	Batch size 4 with gradient accumulation, gradient-norm clipping at 1.0, maximum 120 epochs, early stopping after 30 without improvement	This study
Cross-validation	Evaluation	Nested 5-fold, folds assigned at the patient level, epoch chosen on an inner split, seeds 42, 123 and 7	This study
scikit-learn	Analysis	Clinical comparator (logistic regression), StratifiedKFold, ROC and Brier score	scikit-learn.org
SciPy	Analysis	DeLong test implementation, distribution functions, texture descriptors	scipy.org

Component	Type	Version or configuration	Reference or source
lifelines	Analysis	Cox proportional hazards model, concordance index	lifelines.readthedocs.io
NumPy, pandas, NiBabel	Analysis	Array handling, tabular data, NIfTI input and output	numpy.org; pandas.pydata.org; nipy.org/nibabel
Grad-CAM	Interpretability	Final convolutional block of each endpoint model	Selvaraju et al. [45]
Hardware	Compute	NVIDIA A100 GPU, Google Colab	n/a
